# Advancing the science of organ donor management

**DOI:** 10.1186/s13054-014-0612-z

**Published:** 2014-11-12

**Authors:** Sonny Dhanani, Sam D Shemie

**Affiliations:** Pediatric Critical Care, Children’s Hospital of Eastern Ontario, Ottawa, ON Canada K1H 8L1; Faculty of Medicine, University of Ottawa, Ottawa, ON Canada K1N 6N5; Pediatric Critical Care, Montreal Children’s Hospital, Montreal, QC Canada H3H 1P3; Faculty of Medicine, McGill University, Montreal, QC Canada H3G 2M1

## Abstract

There is an increasing burden of responsibility for intensivists to optimize donation potential after the declaration of brain death in patients with catastrophic brain injury. Best practice for donor management, if present, has been formed on low quality and mainly observational studies or consensus. In particular, research into the use of corticosteroids has shown varied benefit. The specific and limited results of the CORTICOME study are less important than the systematic methodology and the development of rigour in the study of deceased organ donation. Donor management would benefit from continued systematic analysis of current literature, understanding of the physiologic basis for therapy, and further prospective controlled trials. Worldwide collaboration partnerships and funding are needed to optimize the management of deceased organ donation.

Organ donation continues to save lives. There is an increasing burden of responsibility for intensivists to optimize donation potential after the declaration of brain death in patients with catastrophic brain injury. Until recently, organ donor management has been considered a low priority. As such, rigorous research to guide improved organ procurement and graft survival has been limited. Pinsard and colleagues [1] have completed a prospective multi-centre study showing the benefits of low-dose corticosteroids after brain death. Best practice for donor management, if present, has mostly been formed on low quality and mainly observational studies or consensus [2]. It is more evident that the positive impact on organ transplantability when meeting donor management goals is significant [3]. But, benefits and outcomes for individual interventions have been unclear. As a result, focus on donor management and the uptake of best practices in the ICU have been difficult. The systematic approach to research in organ donation management needs to continue with further collaboration and financial support.

Until now, research into the use of corticosteroids has shown varied benefit. The overall quality of studies has been poor with few randomized controlled studies and mostly observational reports with limited patients and high potential for confounding factors. Most of these studies included other hormonal therapies rather than corticosteroids alone [4]. A recent systematic review of the use of corticosteroids in the management of brain dead donors highlighted the low quality and conflicting evidence when identifying outcomes of donor haemodynamics and oxygenation, organ procurement, recipient survival, and graft survival [5]. Studies have shown increased lung utilization with high dose corticosteroids and hormonal therapy [6] while other research has shown minimal negative effects with use of corticosteroids in this group [7]. A large prospective study evaluating the specific effect of corticosteroids was deemed to be warranted.

Pinsard and colleagues should be commended for accomplishing the challenging task of conducting a prospective multi-centre study involving 259 subjects in order to identify the benefits of low-dose corticosteroids after brain death. The authors found that the need for norepinephrine was reduced [1]. Although no benefits were seen on transplantation or graft survival, the reduced dose and duration of vasopressor use may be a clinically significant but limited outcome for intensivists and transplant teams.

Though hypothesized, the mechanism for reduced dose and duration of vasopressors with low dose hydrocortisone is still unclear. Similar results have been found with corticosteroid use in other disease states such as septic shock [8], perhaps as a result of increasing cardiac and vascular sensitivity to catecholamines. Future adoption of best practice in donor management needs to be guided with a deeper understanding of biologic mechanisms and effective interventions. The physiology for organ systems failure after brain death is complex and multifactorial. For example, the documented pro-inflammatory environment in the potential donor [9] has yet to translate into antiflammatory therapies that improve graft function. Optimization of the donor with multimodal approaches including hormonal therapy need to be individualized and based on our increasing knowledge of physiology during the dying process.

Current donor management studies often suffer from paradigm challenges. Traditional ICU interventional studies aim to improve organ function and meaningful outcomes in the patient receiving those interventions. Meanwhile, donor management studies seek to improve organ function *in situ* of another patient, the transplant recipient. Numerous uncontrollable variables independent of donor management impact on transplant outcomes (transplant logistics, surgical procurement, preservation, storage, immunosuppressive therapies, recipient conditions, and so on). While improving transplant outcomes is clearly the most important goal, it may not be the most scientifically valid outcome. The scientific question should first assess whether a specific strategy improves organ function in the brain-dead donor themselves, and then second, assess whether improved donor organ function leads to increased organ utilization and transplant graft survival. Outcomes analysis should change from traditional graft survival to specific physiologic goal-directed targets relevant at the bedside of the donor. We would propose that physiologic outcomes and end organ function, such as haemodynamics, cardiac ejection fraction, oxygenation status, hepatic markers, and renal function, need to be prospectively measured to identify specific benefits of specific donor management therapies.

## Conclusion

The systematic approach to research in organ donation management is evolving slowly and is long overdue. The current work on the use of corticosteroids is an example. The specific and limited results of the CORTICOME study are less important than the systematic methodology and the development of rigour in the study of deceased organ donation. The same must be accomplished for other therapies, individually and combined, including fluid management and haemodynamic supports. In the future, more aggressive, novel, and multimodal interventions need to be studied. Collaboration with transplant research, to identify immunomodulating targets in potential donors, should be pursued. Donor management would benefit from continued systematic analysis of current literature, understanding of the physiologic basis for therapy, and further prospective controlled trials.

For deceased organ donation research to flourish, national and worldwide collaborative partnerships need to be formed as they were developed for other disease states such as sepsis and traumatic brain injury. Appropriate funding must also be committed to prospective research and initiatives. The basis of this collaboration and support would effectively eliminate the 'art' and create a new scientific rigour to the management of deceased organ donation. With increased collaboration and funding, the potential to save lives through optimized organ donation, given the multi-patient recipient impact of each donor, may be greater than that seen with other recent initiatives.
